# Summer School of Brain Mapping and Stimulation Techniques: bridging theory and hands-on experience for young researchers in neurotechnology

**DOI:** 10.25122/jml-2025-1003

**Published:** 2025-08

**Authors:** Livia Livinț-Popa, Hanna Dragoș, Irina Vlad, Victor Dăbală, Vlad Chelaru, Emanuel Ștefănescu, Bianca Crecan-Suciu, Dafin Mureșanu

**Affiliations:** 1 Department of Neurosciences, Iuliu Hațieganu University of Medicine and Pharmacy, Cluj-Napoca, Romania; 2 RoNeuro Institute for Neurological Research and Diagnostic, Cluj-Napoca, Romania; 3 County Emergency Clinical Hospital, Cluj-Napoca, Romania; 4 Faculty of Medicine, Iuliu Hațieganu University of Medicine and Pharmacy, Cluj-Napoca, Romania

## INTRODUCTION

The rapid evolution of neurotechnologies demands innovative educational approaches that bridge theoretical knowledge with hands-on experience. The Summer School of Brain Mapping and Stimulation Techniques, coordinated by Professor Dr. Dafin Mureșanu and Associate Professor Dr. Livia Popa, was developed to address the educational needs of early-career neuroscientists, providing a platform to explore cutting-edge methodologies and collaborate to foster innovation.

As Prof. Dafin Mureșanu noted:

“The Summer School of Brain Mapping and Stimulation Techniques offered an immersive experience into neurotechnologies, integrating theoretical concepts with hands-on training to meet the needs of a rapidly evolving field. Building upon the success of previous editions, this program equips future researchers and medical practitioners with the knowledge and practical skills needed to thrive, shaping the new generation of neuroscientists and driving forward innovation and collaboration.”

Hosted in Cluj-Napoca under the Neurotech^EU^, the European University of Brain and Technology, and Erasmus+ framework, the 2024 Blended Intensive Program (BIP) united immersive cultural experiences with training in electroencephalography (EEG), quantitative electroencephalography (QEEG), eye tracking (ET), and non-invasive brain stimulation, such as transcranial magnetic stimulation (TMS) and transcranial direct current stimulation (tDCS).

Building upon the foundational concepts introduced in our first editorial entitled New Horizons in Neuroscience: The Summer School of Brain Mapping and Stimulation Techniques, which explored the framework of the scientific event, this editorial highlights the practical implementation of the immersive training program.

### Multicultural experience of participants

On the first on-site day, course participants were introduced to Romanian culture and language by specialized faculty members from the Department of Modern Languages Applied to Medicine and the Department of Humanities at the The University of Medicine and Pharmacy Iuliu Hatieganu (UMFIH). Later, the group visited the Turda Salt Mine, a popular tourist destination with historical significance dating back to Roman times, providing students with an opportunity to engage in an intercultural atmosphere.

### The Fundamentals of EEG and QEEG

On September 10^th^, participants delved into the fundamentals of EEG, learning about signal acquisition, montage selection, activation methods (hyperventilation and intermittent photic stimulation), artifacts, and normal and pathological EEG activity from Lecturer Dr. Livia Popa. Further on, Dr. Hanna Dragoș joined, introducing students to QEEG signal preprocessing, covering topics such as data preprocessing, artifact rejection and reduction through methods like complex demodulation, Infinite Impulse Response (IIR) filters, and band-rejection filters. Later in the day, Dr. Victor Dăbală discussed brain mapping concepts, including Independent Component Analysis, Frequency- and Time-Frequency Domain Analysis, and Fast Fourier Transform. The session concluded with discussions on time-domain connectivity. Following the lectures, participants had the opportunity to network with the local team during the intercultural dinner.

### QEEG in practice

The third day was hosted at the The RoNeuro Institute for Neurological Research and Diagnostic, where participants explored brain connectivity and QEEG during a hands-on session. Dr. Hanna Dragoș guided them through concepts of Time-Domain Connectivity (correlation, connectivity matrix, cross-correlation, Granger causality, and transfer entropy) and Frequency-Domain Connectivity (coherence, phase-synchrony, and phase-locking). Later on, all three lecturers from the previous day coordinated teams of participants in QEEG feature extraction. The day ended with a guided tour through the historic center of Cluj-Napoca, allowing students to discover the city's rich culture..

### Eye Tracking and Brain Stimulation Techniques

**Figure 1:**
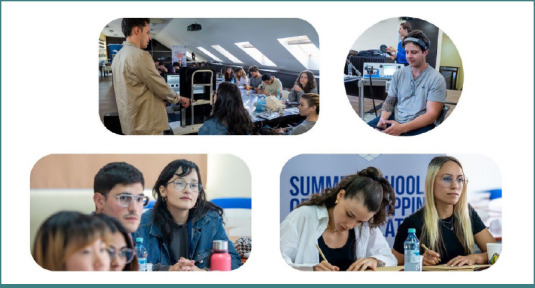
Photos from the sessions. Top row – Dr. Emanuel Stefanescu presenting Eye tracking device and technology and a presentation of tDCS equipment and montage; bottom row – photos taken during the lectures and presentations

September 12^th^ focused on exploring ET in neurology and its clinical applications. After learning the essentials of this exploration method and integration with other technologies, participants engaged in a hands-on workshop led by Dr. Emanuel Ștefănescu (Figure 1) and Dr. Olivia Verișezan Roșu.

The second part of the day included discussions on non-invasive brain stimulation techniques, covering theoretical aspects and applications of TMS (presented by Dr. Georgiana Novăceanu), and tDCS (presented by Dr. Bianca Crecan-Suciu) (Figure 1). The day ended with a hands-on session on both non-invasive neuromodulation methods.

### Wrap-up of the Summer School of Brain Mapping and Stimulation Techniques

Dr. Irina Vlad focused on advanced brain mapping techniques, primarily functional magnetic resonance imaging (fMRI), functional near infra-red spectroscopy (fNIRS), and magnetoencephalography (MEG), emphasizing their crucial role in understanding the brain's functional anatomy and their diverse applications in both research and clinical settings.

### Multisciplinary Collaboration and Teamwork

As a core component of all three editions of the summer school was the group projects, where participants applied and integrated the knowledge and skills they had gained over the seven days to create original research ideas on one of the neurotechnologies.

The five teams presented their group projects to their peers, showcasing their understanding of the program. Each team worked throughout the week to develop a unique project that reflected both the skills they acquired and the ability to collaborate across disciplines. Two teams were awarded for their exceptional work, judged on their comprehension of the lectures, originality, presentation skills, and potential impact. The winning projects were ‘QEEG and Its Use in Predicting Disease Progression in Multiple Sclerosis’, and ‘Combining rTMS and tDCS for Long-Term Effects in Drug-Resistant Major Depressive Disorder’. During the closing ceremony, Dr. Ștefan Strilciuc and Dr. Livia Popa presented the awards, bringing this enriching journey to an end.

“Attending the recent summer school on Brain stimulation techniques in Cluj-Napoca was an invaluable experience that significantly enriched my understanding and skills in the field. As an MSc Neuroscience student, I found the workshops and lectures deeply engaging and directly relevant to my academic and research interests. The opportunity to learn advanced data analysis techniques provided me with practical skills that I can now apply in my research. Collaborating with peers from diverse backgrounds fostered an inspiring environment where I could exchange ideas and gain insights into different perspectives within neuroscience. Overall, this experience not only expanded my technical knowledge but also motivated me to explore new interdisciplinary approaches in my studies. I am truly grateful for this opportunity and would highly recommend it to anyone looking to deepen their understanding of neuroscience research methodologies.”

*Neelam Bhattarai, participant*

## CONCLUSIONS

In a fast-progressing field, it is increasingly important for young specialists to stay connected with the latest technological advancements and work together towards improving science, medical care, and research. Whether future medical physicians, specialists in neurosciences and technology, psychologists, or entrepreneurs, having an all-encompassing knowledge of human brain processes is at the heart of medical and scientific development.

**Figure 2:**
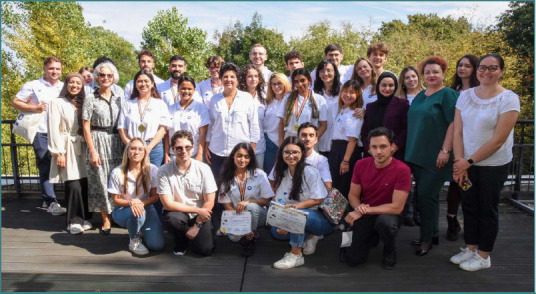
Group photo with lectors and participants from the Summer School of Brain Mapping and Stimulation Techniques

The *Summer School of Brain Mapping and Stimulation Techniques* offered a platform for educating a new generation of medical professionals and scientists in neuroscience and neurotechnology (Figure 2). From learning EEG, QEEG, and ET, to familiarizing with non-invasive brain stimulation techniques (TMS, tDCS), as well as more complex and costly brain mapping neurotechnologies (fMRI, MEG and fNIRS), the blended educational program offered a comprehensive enhanced curriculum completed by hands-on experience, marking the third edition of summer school on neurotechnologies in Cluj-Napoca.

The program’s commitment to multidisciplinary collaboration and education has been pivotal in bridging gaps between scientific fields and driving innovation. As participants worked together to develop original research ideas, reinforcing the core values such as collaboration, critical thinking, and international cooperation. These initiatives might serve as motivation for future multidisciplinary breakthroughs in neuroscience, ultimately contributing to patient care and increased quality of life.

